# Eigenspace generalized sidelobe canceller combined with SNR dependent coherence factor for plane wave imaging

**DOI:** 10.1186/s12938-018-0541-1

**Published:** 2018-08-13

**Authors:** Aácio José Zimbico, Diogo Watchel Granado, Fabio Kurt Schneider, Joaquim Miguel Maia, Amauri Amorin Assef, Nivaldo Schiefler, Eduardo Tavares Costa

**Affiliations:** 1 Electrical Engineering Department (DEEL), Eduardo Mondlane University (UEM), Maputo, Mozambique; 2 Graduate School of Electrical Engineering and Applied Computer Sciences (CPGEI), Federal University of Technology-Parana (UTFPR), Curitiba, PR Brazil; 3 Biomedical Engineering Department of the School of Electrical and Computing Engineering, Biomedical Engineering Centre, State University of Campinas (UNICAMP), Campinas, Brazil

**Keywords:** Minimum variance, Ultrasound imaging, Contrast, Resolution, Coherence factor, Speckle

## Abstract

**Background:**

The eigenspace generalized sidelobe canceller (EGSC) beamformer combined with a signal-to-noise ratio (SNR) dependent coherence factor (CF) is suggested for coherent plane wave compounding (PW) imaging. Conventional CF based methods such as generalized CF and subarray CF can improve the image quality, however, they are not suitable for low SNR. On the other hand, the EGSC CF based approach can introduce improvements in image quality, however, in PW imaging is susceptible to suffer from degradation due to low SNR which leads to a poor image quality. To overcome this limitation, the SNR dependent CF method is suggested for application in such situations due to its ability to control the SNR levels.

**Methods:**

The Field II and the Verasonics ultrasound imaging system with a L11-4v array transducer with a contrast resolution phantom were used to capture the plane wave sequences of simulation and experimental data, respectively. The performance evaluation using full width at half maximum (FWHM), contrast (CR and CNR) and the speckle statistics by using the signal to noise ratio (SNR) complemented by the Rayleigh distribution analysis was performed. In order to evaluate the performance of the $\text {EGSC}_{3}$ (the SNR CF) beamformer, the comparison is done with particular importance to other CF-based approaches such as $\text {EGSC}_{1}$ (the generalized CF) and, $\text {EGSC}_{2}$ (the subarray CF) respectively.

**Results:**

Taking DAS as reference, $\text {EGSC}_3$ showed 30.3 and 39.5% of improvement for $\text {CR(dB)}$ and $\text {CNR}$, respectively, when using experimental data. The proposed method also slightly outperforms the $\text {EGSC}_1$ and $\text {EGSC}_2$ methods for $\text {CR(dB)}$, $\text {CNR}$, and speckle statistics assessment.

**Conclusion:**

The $\text {EGSC}_3$ is, therefore, suitable for CPWC by improving the spatial resolution and contrast while preserving the speckle pattern.

## Background

Medical ultrasonic imaging is a noninvasive and low-cost technology widely used for diagnosis. Several techniques have been recently introduced for medical ultrasonic imaging in addition to the traditional Delay and Sum (DAS) beamforming method. DAS is a non adaptive beamforming method since it applies a fixed weight function (e.g., boxcar and humming) for array data summation. Adaptive weighting is, therefore, expected to result in increased image quality as introduced later. In addition, image reconstruction techniques have been proposed to increase frame rate as in plane wave imaging (PWI) [1, 2]. In PWI the medium is illuminated using all the aperture elements simultaneously to create a plane ultrasound wave. The signal to noise ratio (SNR) is commonly smaller compared to DAS method because PWI uses a single or a few ultrasound plane wavefronts to interrogate the medium in order to create a frame, therefore reducing the ultrasound power per generated frame opposed to the focused firing in DAS. On the other hand, since frame rate is generally limited by the time of flight of the ultrasound signal, PWI can significantly contribute to increase the frame rate of the system [1, 2].

Besides the lower interrogating power, contrary to DAS, in PWI the received signal in all transducer elements is composed by backscattered echoes from many different points from the interrogated medium resulting in lower SNR [1–4] being expected a tradeoff between the frame rate and the image quality in terms of spatial resolution and contrast [1, 2]. The possibility of compounding a set of low-resolution images to get an image with improved spatial resolution and contrast is the basis of spatial compounding (SC) as described by Berson et al. [5] to reduce speckle noise. Additionally, Montaldo [1] proposed a SC method called coherent plane wave compounding (CPWC) for further improvements.

As mentioned earlier, data-dependent weights can be created to also improve image quality in terms of spatial resolution and contrast by maintaining the main lobe while reducing the side lobes [6–9]. One of the most representative adaptive method is the minimum variance (MV) beamformer proposed by Capon [10]. The aim of the MV beamformer is minimizing the output power of a beamformer while keeping the signal undistorted [7, 8] and was previously evaluated into ultrasound imaging [6–8]. In MV-based methods, the covariance matrix (CM) estimate is one of the key steps which determine the performance of the algorithm [7]. Additionally, the adaptive weights are obtained at the cost of additional computation which comes from the need of inverting the CM [6, 7].

In order to obtain a robust CM estimation, Sasso and Cohen-Bacrie [11] proposed the spatial smoothing method, Synnevag et al. [7] introduced the diagonal loading (DL) technique and, Asl and Mohammadzadeh [12] introduced forward–backforward method. Likewise, Mehdizadeh [13] proposed the Eigenspace-based MV (EMV) beamformer to improve the image quality. The EMV consists of projecting the MV weights onto the signal subspace where a considerable part of the noise is removed from the received data. The signal subspace is constructed from the eigen decomposition of the CM. This procedure consists of sorting the eigenvalues of CM such that the eigenvectors associated to the larger eigenvalues determine the basis for the signal subspace [13, 14].

Another robust representation of MV beamformer is the generalized sidelobe canceller (GSC) beamformer proposed by Applebaum and Chapman [15] and later popularized by Jim and Griffiths [16] in the area of array signal processing. The GSC has been implemented in ultrasound imaging and compared to DAS and MV beamformers [17–20]. Similarly to EMV compared to MV, projecting the GSC weight onto the signal subspace lead to the eigenspace-based GSC (EGSC) beamformer implemented by Aliabadi et al. [19] resulting in improved image quality at increased computational cost when compared to GSC beamformer.

A coherence factor (CF) may also be combined into adaptive beamformers such as MV and was previously used for aberration correction and sidelobe suppression in ultrasound imaging [21].

Li and Li [22] have proposed the implementation of generalized CF (GCF) approach in MV beamformer to improve imaging resolution by protecting the main lobe. To improve the GCF performance, Zhau et al. [23] have suggested the subarray CF (SCF) approach. The SCF has optimized the performance of the EMV beamformer [23] at the cost of increased computational complexity. As in GCF, the SCF results from a combination of the incoherent sum and the coherent sum of signal energy [24, 25]. To address the limitations of GCF and SCF, Wang et al. proposed the SNR dependent CF (SNR-CF). In the SNR-CF method, a weighting scaled factor determined by the sigmoid function is combined with the coherent and incoherent summations of signal energy in order to control local levels of SNR [23]. Due to the SNR-CF features, the effects of signal cancellation at low SNR were minimized while the effects of self-cancellation were controlled at high SNR, which may introduce improvements in spatial resolution and contrast [24].

Considering SNR-CF based method is suitable for applications in signals with low SNR and recognizing that PWI typically presents low SNR [23], the SNR-CF method could aggregate some benefits [24, 25] into PWI-based methods. In this work, we suggest a combination between the EGSC with SNR dependent CF ($\text {CF}_3$) method to form the ($\text {EGSC}_3$) beamformer. For comparison, the GCF ($\text {CF}_1$) is also combined with EGSC to obtain the ($\text {EGSC}_1$) beamformer while the SCF ($\text {CF}_2$) combines with EGSC to form ($\text {EGSC}_2$) beamformer. The tests were performed using simulated and phantom data.

## Methods

The coherent plane wave compounding principle is firstly presented followed by evolved beamforming methods including different adaptive techniques such as MV, EMV, GSC, EGSC, $\text {EGSC}_1$, $\text {EGSC}_2$ and $\text {EGSC}_3$. These techniques are the basis for the proposed SNRCF-based EGSC method and are later used for beamforming performance assessment.

### Beamforming methods

#### Coherent plane wave compounding

**Fig. 1:**
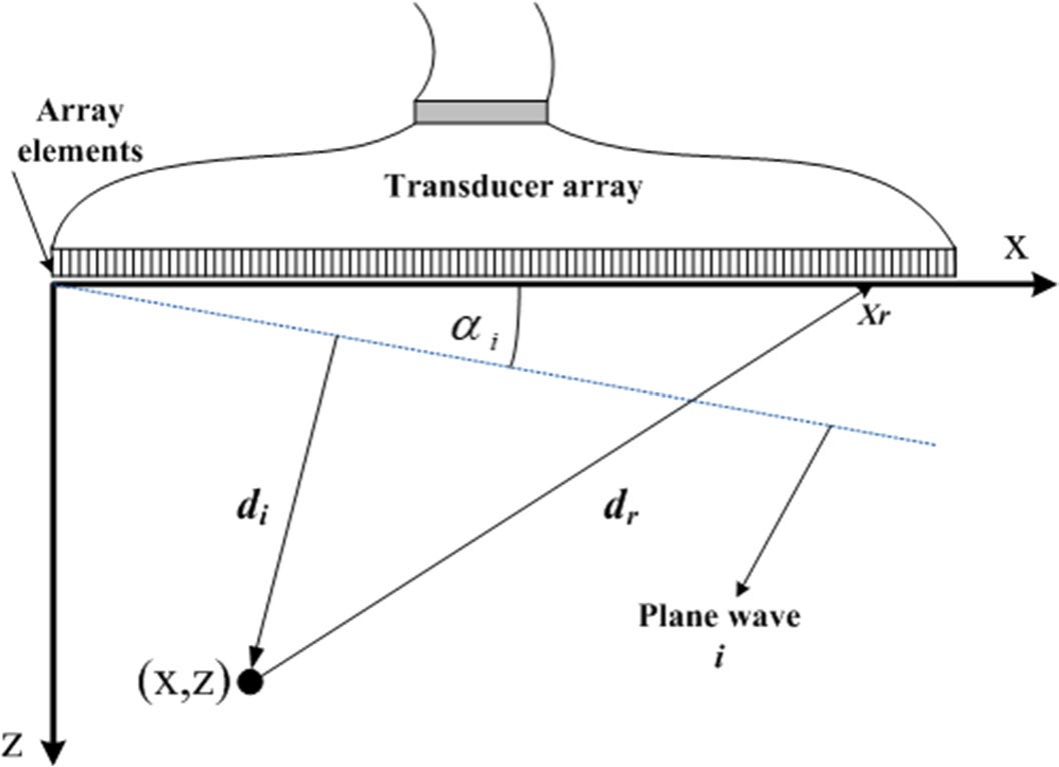
Geometrical entities of the imaging system

In Eq. (1), we assume that a sampled time series $\text {X}(k)$ is represented in a measurement grid as $\text {X}_{\text {CPWC}}(x,z)$ whose geometrical entities are depicted in Fig. 1, is measured for each element in a linear array where, M ($r=1,\ldots ,M$) is the element number, N ($i=1,\ldots ,N$) is the number of wave emissions, $\text {w}$ represents the receive apodization window, *u* is the angular apodization, $h_{ir}$ is the impulse response of element *r* to plane wave *i*. For each time sample, $\tau _i=(di+dr)/c$ represents the total travel time for *i*th wave where $d_i=zcos\alpha _i+xsin\alpha _i$, is the distance from wave emission to point (x, z), *dr* is the distance from point (x, z) to the receive *r*th array element and, c is the sound speed set to be 1540 m/s [26].

1 $$\begin{aligned} \text {X}_{\text {CPWC}}(x,z)= \sum _{\text {r}=1}^{{\text {M}}} \text {w}(\text {x}_{r}) \sum _{i=1}^{\text {N}} u(\alpha _i) h_{ir}\big (\tau _i\big ) \end{aligned}$$

The angle sequence $\alpha$ can be formulated as in Eq. (2) where, $\lambda$ is the wavelength of the system and, A represents the probe aperture [26].

2 $$\begin{aligned} \alpha _i=\psi \big (i,N,\lambda ,A\big ), \varvec{\text { }} \quad i=1,\ldots ,\text {N} \end{aligned}$$

Individual signals collected in Eq. (1) follow the array geometry representation in Fig. 1 and can be represented in the form of a 2-D data matrix X(k) as in Eq. (3), where $k=1,\ldots ,K$. In this context, *K* represents the total amount of time samples recorded in each array element,

3 $$\begin{aligned} \text {X}(\text {k}) = \begin{bmatrix} \text {x}_{\text {1,1}}(\text {k})&\text {x}_{\text {1,2}}(\text {k})&\cdots&\text {x}_{\text {1,M}}(\text {k})\\ \text {x}_{\text {2,1}}(\text {k})&\text {x}_{\text {2,2}}(\text {k})&\cdots&\text {x}_{\text {2,M}}(\text {k})\\ \cdots&\cdots&\ddots&\cdots \\ \text {x}_{\text {N},\text {1}}(\text {k})&\text {x}_{\text {N},\text {2}}(\text {k})&\cdots&\text {x}_{\text {N},\text {M}}(\text {k}) \end{bmatrix} \end{aligned}$$

where, $\text {x}_{\text {ir}}(\text {k})$, is the received signal in element *r* for the *i*th wave emission. In CPWC, the beamformer output Eq. (4) is obtained by averaging the data in Eq. (3) as follows:

4 $$\begin{aligned} \text {z}(\text {k})= \frac{1}{\text {MN}}\sum _{i=1}^{\text {N}}\sum _{r=1}^{\text {M}} \text {x}_{ir}(k) \end{aligned}$$

In CPWC with a sequence of tilted plane waves, the angular and the receive apodization effects are taken into account in order to form Eq. (4). Additionally, in the conventional adaptive beamforming, the receive apodization window is placed by the data-dependent weight [4].

#### Minimum variance

In MV beamformer (BF) the weight is found by minimizing the Power ($w^{\text {H}}Rw$) of the BF output (s.t) subject to the directional constraints as follows:

5 $$\begin{aligned} \text {w}=\text {argmin} \big \{\varvec{\text {w}^\text {H}\text {R}\text {w} } \big \}\,\,\rm{s.t}\, \varvec{\text {w}^\text {H}\text {a}} = \varvec{1} \end{aligned}$$

where $\text {R}=E\big \{\text {XX}^{\text {H}}\big \}$, $E[\cdot ]$ is the expectation operator and, $[\cdot ]^{\text {H}}$ is the Hermitian operator, respectively.

The steering vector $\varvec{\text {a}}$ (i.e., a vector of ones) follows a structure designed for the cases when time-delay steering is used such that the desired signal is approximately in phase at the steered outputs [7, 16]. The solution of the minimization problem in Eq. (6) using the Lagrangian multipliers [7, 9] is:

6 $$\begin{aligned} \mathbf{w}_\mathbf{MV}=\frac{\mathbf{R}^{-1} \mathbf{a} }{\mathbf{a}^{\text {H}} \mathbf{R}^{-1} \mathbf{a} } \end{aligned}$$

In accordance with the presented in Eq. (5), the MV BF aims to minimize the output power of the output signal while keeping it undistorted [7, 9]. In practice, the covariance matrix (CM) R Eq. (7) is estimated from the data by averaging in the temporal domain, averaging in the spatial domain or, the combining temporal and spatial domains [7].

While the former is performed taking into account $F=2T+1$, where *T* is the number of temporal samples used in covariance matrix (CM) estimation, the latter considers the $G=M-L+1$ overlapping subarrays $\text {X}_{\text {l}}(k)$ whose length is limited to ($L\le M/2$) [7].

Subarray averaging aims to decorrelates the coherence between ultrasound signals and consists of dividing the dataset into overlapped submatrices as follows: $\text {X}_{\text {l}}({{\text {k}}}{\tiny })=[\mathbf x _{\text {l}}(\text {k}), \mathbf x _{\text {l}+1}(\text {k}),\ldots ,\mathbf x _{\text {l+L}-1}(\text {k})]^{\text {T}}$ and, $(\cdot )^{\text {T}}$ denotes the transpose. After performing this procedure, the CM estimate *R*, can be subject to diagonal loading (DL) method in order to get a robust estimate [7]. The formulation of CM estimate in Eq. (7) combines the previous aspects as follows [7]:

7 $$\begin{aligned} \text {R}=\frac{1}{F} \frac{1}{G} \sum _{\text {k}=-\text {T}}^{ \text {T}}\sum _{\text {l}=1}^{\text {G}}\text {X}_\text {l}(\text {k})\text {X}_\text {l}(\text {k})^\text {H}+\mu I. \end{aligned}$$

where $\mu =1/(\Delta \text {L})\text {tr} \big (\text {R}\big )$, R corresponds to the first term of Eq. (7), $\text {tr}$ is the trace and, $\Delta$ stands for a DL factor [7, 27]. The value of the DL factor $\Delta$ adopted for data processing is included in Table 2.

In minimum variance (MV) BF, the output in Eq. (8) is obtained by applying a set of weights to the received signals as follow.

8 $$\begin{aligned} \text {z}_\mathbf MV (\text {k})= \frac{1}{G}\sum _{l=1}^{\text {G}} \mathbf w _\mathbf MV \text {X}_l(k) \end{aligned}$$

#### Eigenspace minimum variance

In the EMV beamformer, the eigen analysis of the CM is performed in order to construct the signal and the noise subspaces as in Eq. (9)[13]:

9 $$\begin{aligned} \text {R}=\text {U} \Lambda \text {U}^{\text {H}} =\text {U}_{\text {s}} \Lambda _{\text {s}} \text {U}^{\text {H}}_{\text {s}}+\text {U}_{\text {n}} \Lambda _{\text {n}} \text {U}^{\text {H}}_{\text {n}}=\text {R}_{\text {s}}+\text {R}_{\text {n}} \end{aligned}$$

where $\text {R}_{\text {s}}$ and $\text {R}_{\text {n}}$ are the signal and the noise the CM , $\Lambda =\text {diag} \big [\lambda _1, \lambda _2,\ldots , \lambda _L\big ]$ are the eigenvalues in decreasing order such that $\lambda _1 \ge \lambda _2\ge ,\ldots , \ge \lambda _L$ and $\text {U} =\big [\text {v}_1,\text {v}_2,\ldots , \text {v}_\text {L}\big ]$ are the eigenvectors $\text {v}_i$ ($\text { i}=1,2,\ldots , \text {L}$ ) of $\lambda _i$. The subspace estimation is obtained based on a threshold $(_\text {th})$ such that $\text {U}_{s} =\big [\text {v}_1, \text {v}_2,\ldots , \text {v}_\text {th}\big ]$ is the basis for the signal subspace (i.e. for the largest eigenvalues) and $\text {U}_{\text {n}} =\big [\text {v}_{\text {th}+1}, \text {v}_{\text {th}+2},\ldots , \text {v}_\text {L}\big ]$ makes the basis for the noise subspace for the remaining (smaller) eigenvalues [13]. Moreover, the number of eigenvalues used to construct the signal subspace is data dependent and varies in accordance with the amount of signal energy attained at a specific point. The largest eigenvalues used to identify the eigenvectors for constructing the signal subspace are obtained as follows: $\lambda _i\ge \alpha _{\text {th}} \lambda _1$, for ($\alpha _{\text {th}} > 1$) or $\lambda _i \le \beta _{\text {th}} \lambda _L$ for ($\beta _{\text {th}} < 1$), where the $\lambda _i$ is the eigenvalue corresponding to eigenvector used to construct the signal subspace [13, 19, 28].

The EMV weight $\text {w}_{\text {EMV}}$ is found by projecting the MV weight $\text {w}_{\text {MV}}$ onto the signal subspace ($\text {P}_{\text {s}}=\text {U}_{\text {s}}\text {U}_{\text {s}}^\text {H}$) as in Eq. (10) [13].

10 $$\begin{aligned} \mathbf w _\mathbf EMV = \mathbf U _\mathbf s \mathbf U _\mathbf s ^\mathbf{H } \mathbf w _\mathbf{MV } \end{aligned}$$

#### The generalized side lobe canceller

The weight of GSC BF is formulated by minimizing the output power of adaptive beamformer [15, 16, 19]. The GSC weight in Eq. (13) is found by partitioning the optimal weight of Eq. (6) in terms of orthogonal (adaptive) branch as in Eq. (11) and the nonadaptive branch as in Eq. (12), respectively [16]:

11 $$\begin{aligned} \text {w}_{\text {p}}= (\text {B}^{\text {H}} \text {R} \text {B})^{-1}\text {B}^{\text {H}} \text {R} \text {w}_{\text {q}}=2\text {H}^{-1}\text {B}^{\text {H}} \text {R} \text {w}_{\text {q}} \end{aligned}$$

12 $$\begin{aligned} \text {w}_{\text {q}}= (\text {aa}^{\text {H}})^{-1}\text {a} \end{aligned}$$

13 $$\begin{aligned} \text {w}_{0}=\text {w}_{\text {q}}-\text {Bw}_{\text {p}}=\text {w}_{\text {GSC}} \end{aligned}$$

where $\text {H}=2\text {B}^{\text {H}} \text {R} \text {B}$ is the Hessian matrix (HM) [17], $\text {B}$ the blocking matrix (i.e., Eq. 14) of dimensions $\text {L} \times {\text{( }L-1)}$ and the steering vector $\varvec{\text {a}}$ with $\text {1} \times {\text {L}}$ such that: $\text {a}^{\text {H}}\text {B}=0$ [19, 27].

14 $$\begin{aligned} \text {B} = \begin{bmatrix} 1&-1&0&0&\cdots&0\\ 0&1&-1&0&\cdots&0\\ \vdots&\vdots&\vdots&\vdots&\vdots&\vdots \\ 0&0&\cdots&0&1&-1 \end{bmatrix}^{\text {T}} \end{aligned}$$

### The eigenspace GSC

The EGSC BF is found by projecting $\text {w}_{\text {GSC}}$ onto the signal subspace [19, 27].

15 $$\begin{aligned} \mathbf w _\mathbf EGSC = \mathbf U _\mathbf s \mathbf U _\mathbf s ^\mathbf{H } \mathbf w _\mathbf{GSC } \end{aligned}$$

#### Generalized coherence factor (GCF) and subarray-based coherent factor (SCF)

The ratio between the coherent sum (CS) and incoherent sum (IS) is termed as coherence factor (CF) [22]. The GCF or $\text {CF}_1$ is formulated by combining the CS and IS as follow [24]:

16 $$\begin{aligned} \mathbf{CF}_1=\frac{\mathbf{CS}}{\mathbf{IS}}= \frac{\big [\frac{1}{\mathbf{L}} \sum _{i=1}^{L} \mathbf{x}_i (\mathbf{k})\big ]^2 }{\frac{1}{\mathbf{L}} \sum _{i=1}^{\mathbf{L}} [\mathbf{x}_i^2 (\mathbf{k})] } \end{aligned}$$

Accordingly, the SCF of $\text {CF}_2$ is formulated as in Eq. (17) [23, 24].

17 $$\begin{aligned} \mathbf{CF}_2=\frac{\mathbf{CS}}{\mathbf{CS}+\frac{1}{\text {L}}(\mathbf{IS}-\mathbf{CS})}. \end{aligned}$$

#### The SNR dependent CF

One problem of using coherent factors is that the imaging system can give suboptimal results because in such applications the noise lowers the signal coherence and thus the noise suppression capabilities of other CF-based methods are compromised. Different to the $\text {CF}_1$ and $\text {CF}_2$ methods, the $\text {CF}_3$ method takes into account the local SNR in the CF formulation so that contrast of the imaging system can be restored despite the low SNR [23, 24]. In practice, such local SNR values are averaged for half or one wavelength to improve the robustness [24]. In order to compute the weighting value $\eta (\text {SNR})=\eta (\text {P}_\text {s},\text {P}_\text {n})$ the reference ratio is formulated as follows [24]:

18 $$\begin{aligned} \eta ({{\text {P}}_{\text {s}}},{{\text {P}}_{\text {n}}}) = \frac{{{\mathbf {L}} - 1}}{{2{\mathbf {L}}}}\left\{ {1 - {\mathbf {tanh}}\left[ {{\alpha _{{\text {snr}}}}\left( {\frac{{{{\mathbf {P}}_{\text {s}}}}}{{{{\mathbf {P}}_{\text {n}}}}} - {\beta _{{\text {snr}}}}} \right) } \right] } \right\} + \frac{1}{{\mathbf {L}}}\end{aligned}$$

where L represents the subarray length.

19 $$\begin{aligned} \mathbf CF _3 =\frac{\mathbf{CS}}{\mathbf{CS}+\eta (\mathbf{SNR})(\mathbf{IS}-\mathbf{CS})} \end{aligned}$$

**Fig. 2:**
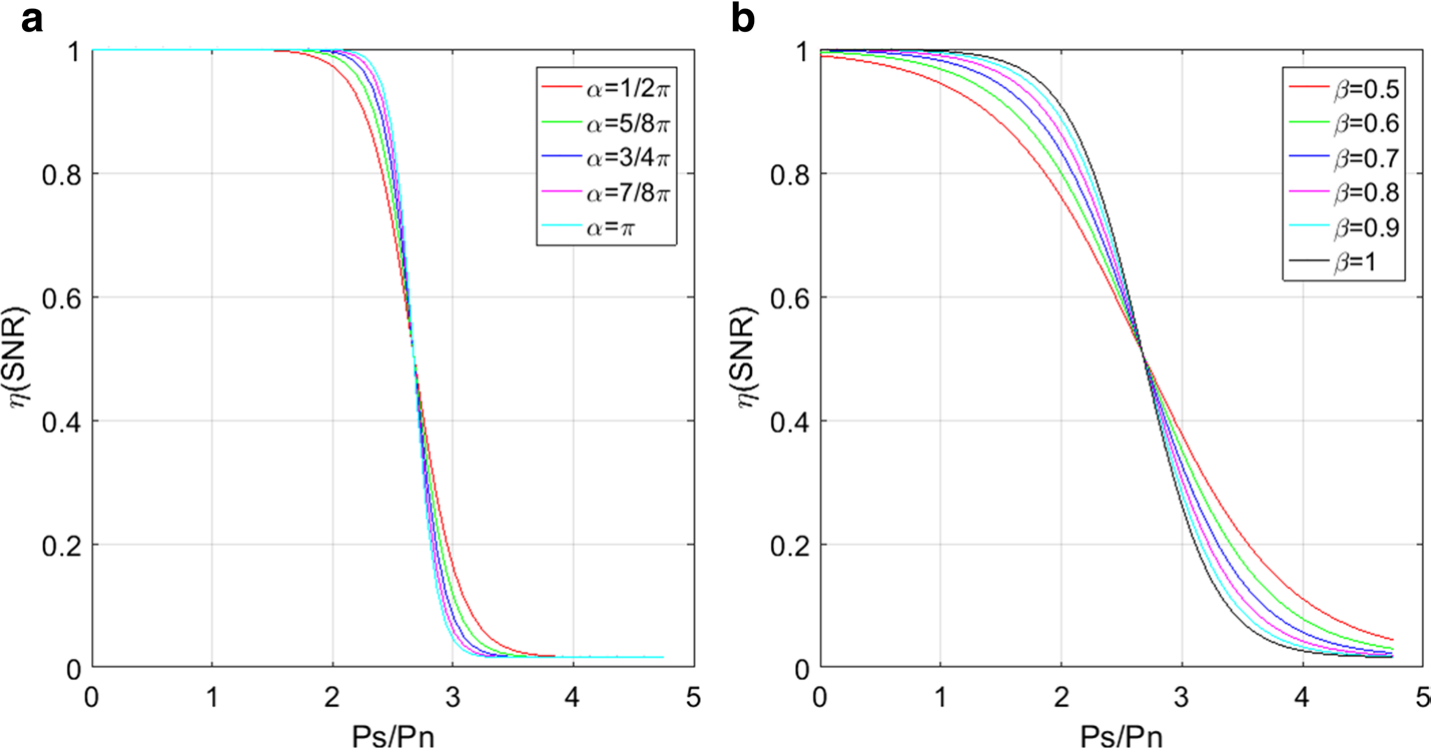
A sigmoide structure Eq. (19) for different values of **a** $\alpha _\text {snr}$ and **b** $\beta _\text {snr}$, respectively

The values defined on Eq. (18), range from 1/L to 1 allowing control the SNR levels while performing adaptive processing avoiding signal cancelation at low SNR while controlling self-cancelation at high SNR. A sigmoid structure for different values of $\alpha _\text {snr}$ (see Fig. 2a) and $\beta _\text {snr}$ (see Fig. 2b) is depicted. For data processing, values of $\alpha _\text {snr}$ and $\beta _\text {snr}$ are presented in Table 2.

#### Proposed SNR CF-based EGSC

In this work, the core of the implementation of the CF-based methods is the EGSC beamformer. More emphasis is given to the combination with the SNR CF ($\text {CF}_3$) since it appears to offer better performance over low SNR data [23, 24]. Moreover, the EGSC ideally provides the better performance than the DAS, MV, EMV and GSC [27] beamformers, so that the CF-based methods are suggested to be combined with the EGSC beamformer to obtain the EGSC-CF based beamformers. In addition, the (SNR) coherence factor (CF) method ($\text {EGSC}_3$) is then compared with the following categories of CF based methods: the GCF ($\text {CF}_1$) and the SCF ($\text {CF}_2$) [23, 24], respectively.

The EGSC weight ($\mathbf w _\mathbf{EGSC }$) can be combined with $\text {CF}_1$, $\text {CF}_2$ and $\text {CF}_3$ to form the EGSC-GCF, EGSC-SCF and EGSC-SNR-CF, as presented in Eqs. (20), (21) and (22), respectively.

20 $$\begin{aligned} \mathbf{w}_\textbf{EGSC-1}=\mathbf{CF}_1 \mathbf{w}_{\mathbf{EGSC}} \end{aligned}$$

21 $$\begin{aligned} \mathbf{w}_\textbf{EGSC-2}=\mathbf{CF}_2 \mathbf{w}_{\mathbf{EGSC}} \end{aligned}$$

22 $$\begin{aligned} \textbf{w}_\mathbf{EGSC-3}= \mathbf{CF}_3 \mathbf{w}_{\mathbf{EGSC}} \end{aligned}$$

#### Implementation procedure

The key steps for implementation procedures are summarized in Table 1. This process is repeated for each imaging point to get the final image which is finally displayed in dynamic range of 60 dB.

**Table 1:** Review of the implementation procedures of the proposed beamformer algorithm

I.	Signals in Eq. (1) are arranged to form the 2-D matrix data representation in Eq. (3). Different firing events correspond to different steering angles thus, the calculated time delay is different. For each emission, the RF data are collected and combined with the calculated time delays to get the CPWC signals
II.	Estimate CM for the receive aperture. The spatial smoothing, the temporal averaging and the DL approach are performed in order to obtain a robust estimate of CM as in Eq. (19)
III.	Combine the data CM estimate with the steering vector a to compute the MV beamformer weight as in Eq. (6)
IV.	Compute the GSC beamformer weight in Eq. (13) by combining the adaptive Eq. (11) and the nonadaptive braces Eq. (12), respectively
V.	Perform the eigen decomposition of CM in order to construct the signal subspace in Eq. (9) and, compute the EGSC weight using Eq. (15)
VI.	Compute the weights for the CF based methods $\text {EGSC}_1$, $\text {EGSC}_2$ and $\text {EGSC}_3$ in Eqs. (16)–(19) using Eqs. (15), (16), (15)–(17) and (15)–(19), respectively
VI.	Using Eq. (8) apply Eqs. (20)–(22) to compute the beamformer output for the proposed methods

### Evaluation metrics

The evaluation metrics for resolution analysis were FWHM and PSL while CR, CNR, SNR, and the theoretical Rayleigh distribution (TRD), were used for image contrast and speckle assessment as follow.

#### Full-width half maximum (FWHM) and peak side lobe levels (PSL)

The FWHM [29] and the PSL [27] values are computed for evaluation of spatial resolution and interference. The former defines the beam width of the main lobe at − 6 dB while the latter defines the level of the first side lobe level for evaluating the interference and noise suppression abilities of a beamformer. Normally, the evaluation of FWHM and PSL is complemented by plots of normalized profiles and, improvements are presented by the narrower beam width and lower PSL [29].

#### Contrast ratio (CR), contrast to noise ratio (CNR) and, signal to noise ratio

For anechoic cyst and hyperechoic target to background evaluation, the contrast ratio (CR) and contrast to noise ratio (CNR) was assessed using Eqs. (24) and (25) [23]. In this context, an increase of CR and CNR indicates improvements in image quality.

Additionally, the speckle statistics was assessed using the SNR as in Eq. (26) and the histogram of the Rayleigh distribution (RD) Eq. (26) [7, 23, 29].

23 $$\begin{aligned} \text {CR} = {\left| {\Phi _{cyct} - \Phi _{bck} } \right| } \end{aligned}$$

24 $$\begin{aligned} \text {CNR} = \frac{{\left| {\Phi _{cyct} - \Phi _{bck} } \right| }}{{\sqrt{\sigma _{cyst}^{2} + \sigma _{bck}^{2} } }} \end{aligned}$$

25 $$\begin{aligned} \text {SNR} = \frac{{ {\Phi } }}{{ \sigma }} \end{aligned}$$

where ${\Phi }_{cyst}$, is the mean amplitude (before log-compression) in the cyst and ${\Phi }_{bck}$ is the mean amplitude in the speckle, ${\sigma _{cyst}^{2} }$ variances of intensities inside cyst and ${\sigma _{bck}^{2} }$ in the background, respectively. In Eq. (25), ${\Phi }$ and $\sigma$ respectively the mean value and standard deviation. The fully developed speckle is approximated by the RD which is expressed by the probability density function Eq. (26) for the envelope amplitude in an image as follows [29]:

26 $$\begin{aligned} p(\Phi )=\bigg (\frac{2\Phi }{\overline{\Phi }^2}\bigg ) \mathbf exp \bigg (\frac{-\Phi }{\overline{\Phi }^2}\bigg ) \end{aligned}$$

where $\overline{\Phi }^2$ is the mean of the squared amplitude.

**Fig. 3:**
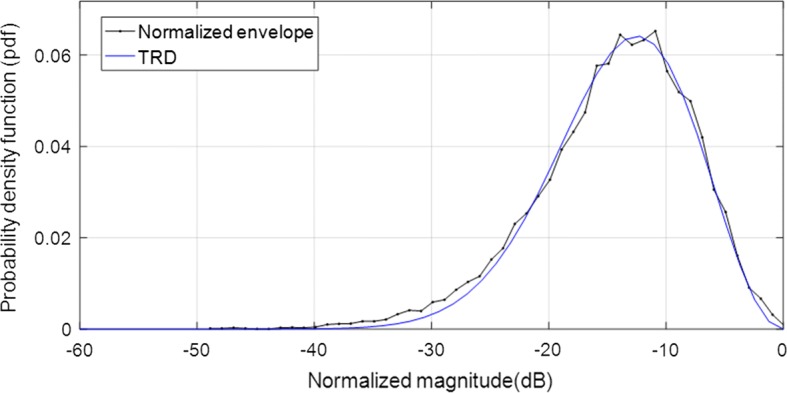
Ilustration of theoretical pdf for speckle assessment

Since the speckle pattern of DAS beamformer follows closely the RD, the TRD with $\text {SNR}=1.91$ [7, 29] was included for illustration in Fig. 3 where the TRD together with the histogram of echo brightness (amplitudes) for simulated data are presented.

One can examine if the speckle pattern of the beamformed data follows the RD by performing the hypothesis test (K-test 5% significance). The test is decided when the computed p-value is compared with the maximal extreme of the significance interval [23].

Furthermore, a decrease of the amplitude level of the histogram indicates the speckle degradation effect [29].

### Simulations and experiments

In order to test the applicability of the proposed method in CPWC imaging, we used the simulated and phantom dataset. The simulated data were acquired using simulated phantoms in Field II program for individual point scatters and the circular anechoic cysts [30]. A 128-element linear array transducer model L11-4v with center frequency of 6.25 MHz and fractional bandwidth of 60% was simulated. A two-cycle sine wave excitation pulse at the central frequency and sampling frequency of 40 MHz were used.

A set of point targets were simulated in a homogeneous medium. The point target simulation phantom with fifty scattering points was created. The points were laterally distributed (x = 0 mm, x = ±5 mm and x = ±10 mm) from an axial depth of (z = 20 mm) to (z = 45 mm) spaced 5 mm apart from each other.

Two circular anechoic cysts of 5 mm radius were simulated in a speckle pattern. The anechoic cysts with a diameter of 10 mm centered at lateral positions ($x =+10$) and ($x= -10$ mm) at the axial position of (z = 42.5 mm) were placed in a simulated homogenous media with 80,000 scattering points.

**Fig. 4:**
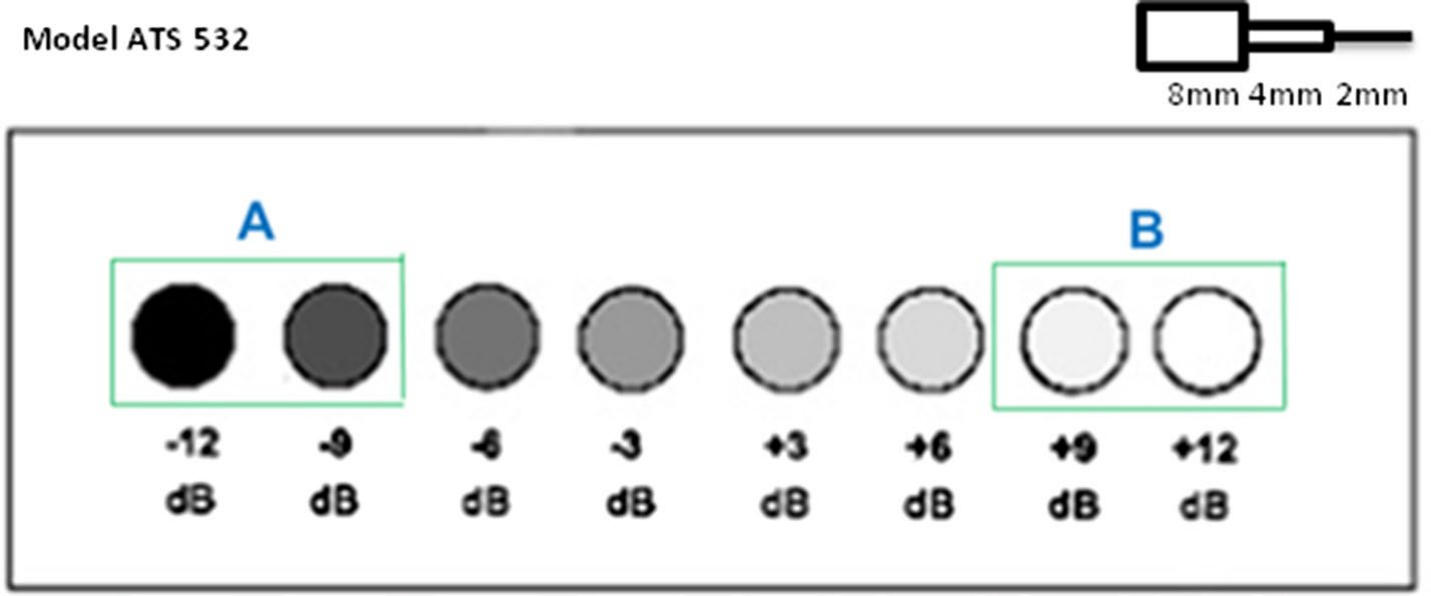
The contrast resolution phantom containing cysts with different contrast values

For experiments, the contrast detail resolution ultrasound phantom ATS model 532A containing several circular anechoic cysts and hyperechoic targets with different values of contrast was used. In accordance with Fig. 4, we decided recording data positioned at 8 mm diameter along the phantom.

Data corresponding to a pair of closely set anechoic cysts (label A) with − 12 and − 9 dB and another pair of hyperechoic cysts (label B) with + 9 and + 12 dB (see Fig. 4) were acquired. The background (speckle) and the regions inside the cysts have been used as the reference to compare the performance of the different beamformers.

A 128 element linear array transducer working at 6.25 MHz central frequency and pitch of 0.3 mm was used. For data processing, the sampling frequency was set to be 25 MHz. Verasonics imaging system using an L11-4v (Verasonics Ltd, Kirkland-WA, USA) transducer was used to acquire data with a sampling frequency of 25 MHz and transducer center frequency of 6.25 MHz.

For both simulation and the experimental studies, all the array elements were used to emit the plane waves and record the RF data. Furthermore, either in emission or the reception, the acquisition system was not apodized.

Additionally, for simulation and the experiments, the plane waves were emitted with different steering angles for multiple scanning. All the transmitting steering angles were set to range from $-5^{\circ }$ to $5^{\circ }$ (21 angles with an interval of $0.5^{\circ }$) and, the f-number of 1.75 was used.

For adaptive processing, a subarray of L = M/2 = 128/2 = 64 elements was used for spatial smoothing. For CM estimate, we used one temporary sample (i.e., $F=2T+1=2 \times 0+1=1$) and $\Delta$ of 0.01 [7]. For signal subspace construction, a $\alpha_{\text {th}}$ of 5 or $\beta_\text {th}$ of 0.05 was adopted [19, 28]. For $\text {CF}_3$ calculation, the weight $\eta (\text {SNR})$ in Eq. (18) was computed using a $\alpha _\text {snr}$ of $\pi$ and $\beta _\text {snr}$ of 0.5, respectively [24]. All the imaging parameters are presented in Table 2.

**Table 2:** Important parameters for simulated and experimental for acquisition and data processing

Imaging parameters	Simulation	Experimental
Central, sampling frequencies (MHz)	6.25, 25	6.25, 40
Transducer model	L11-4v	L11-4v
Number of elements (NE)	128	128
NE for emission, reception	128, 128	128, 128
Subarray length (L)	64	64
Sound speed (m/s)	1540	1540
Fractional bandwidth (%)	60	60
Pitch (mm)	0.3	0.3
F number	1.75	1.75
Range, angles, gap	$-5^{\circ }$ to $5^{\circ }$, 21, $0.5^{\circ }$	$-5^{\circ }$ to $5^{\circ }$, 21, $0.5^{\circ }$
Data acquiring	From field II	CRP (ATS)
Diagonal loading	$\Delta =0.01$	$\Delta =0.01$
Subspace construction	$\alpha _{\text {th}}=0.05$	$\alpha _{\text {th}}=0.05$
SNR-CF weight param	$\alpha _\text {snr}=\pi$, $\beta _\text {snr}=0.5$	$\alpha _\text {snr}=\pi$, $\beta _\text {snr}=0.5$

*CRP* contrast resolution phantom (model: ATS)

All the beamformers were implemented and tested using MatlabR$^{\circledR }$2016b using a Intel T6600 at 2.2 GHz, 4G byte RAM and 64-bit Windows R$^{\circledR }$ 10.

## Results and discussion

The simulated and phantom dataset was used to evaluate the performance of the proposed beamformers.

### Simulation: point targets

**Fig. 5:**
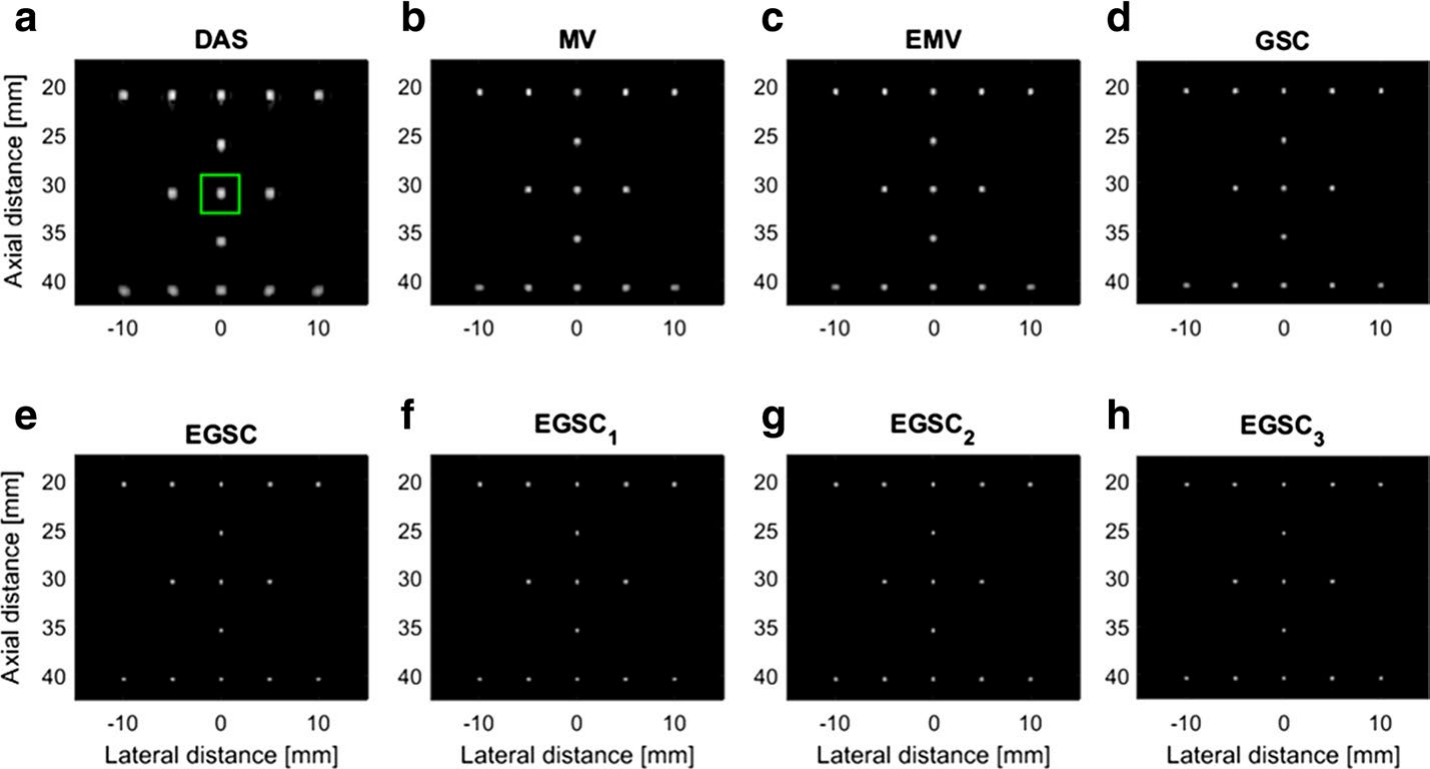
The beamformed responses for the simulated targets using different techniques: **a** DAS, **b** MV, **c** EMV, **d** GSC, **e** EGSC, **f** $\text {EGSC}_1$, **g** $\text {EGSC}_2$ and **h** $\text {EGSC}_3$, respectively. The corresponding lateral profiles are depicted in Figs. 6 and 7, respectively

**Fig. 6:**
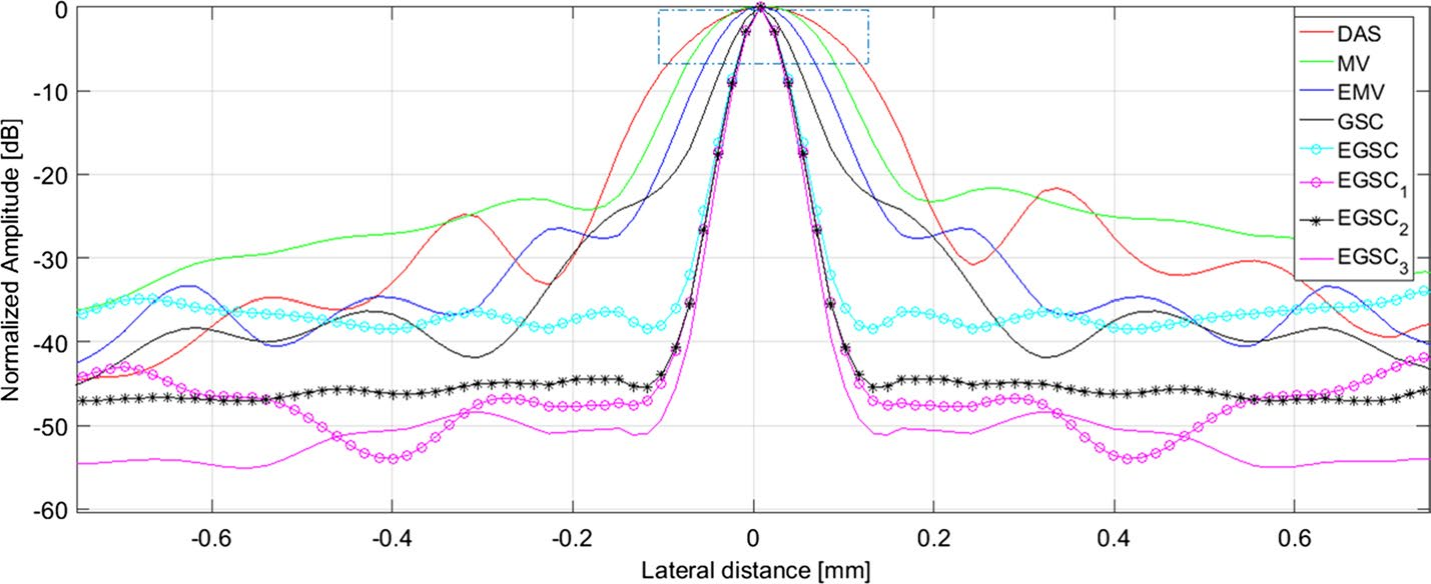
The normalized responses (see Fig. 5) for the simulated targets at different (x, z) positions using different beamformers: (a) DAS, (b) MV, (c) EMV, (d) GSC, (e) EGSC, (f) $\text {EGSC}_1$, (g) $\text {EGSC}_2$ and (h) $\text {EGSC}_3$, respectively. The dot dashed plots are well visualized in the figure

Fifteen point targets were simulated in order to test the performance of different beamformers as shown in Fig. 5 with a dynamic range of 60 dB. The point targets are respectively located at depths of 20 mm, 25 mm, 30 mm, 35 mm and 40 mm. The lateral profiles for FWHM and PSL assessment are shown in Fig. 6 using the point target highlighted in green box at (x, z) = (0, 30) mm in Fig. 5a as reference.

**Fig. 7:**
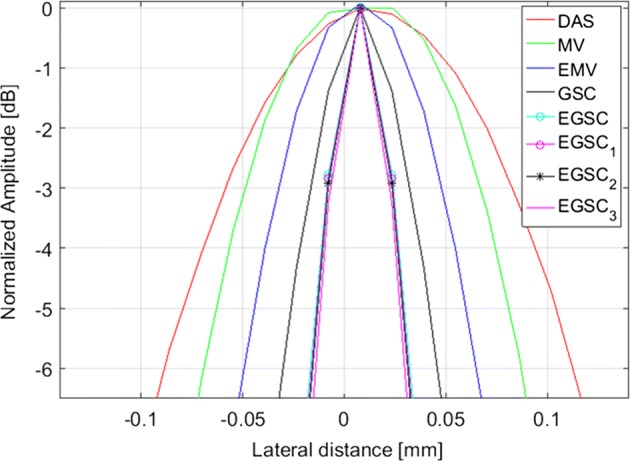
The normalized responses for the simulated targets (see Fig. 5) at different (x, z) positions using different beamformers: (a) DAS, (b) MV, (c) EMV, (d) GSC, (e) EGSC, (f) $\text {EGSC}_1$, (g) $\text {EGSC}_2$ and (h) $\text {EGSC}_3$, respectively. The plots highlighted by the dot-dashed box in Fig. 6 are shown with improved visibility

Figure 6 presents the improvements introduced by adaptive techniques over the traditional DAS method. Figure 7 presents the 0 to −  6 dB region in order to better the visualize the lateral resolution improvement.

From displayed images, it can be seen that DAS has a wide main lobe and higher side lobes compared to the MV beamformer. The EMV provides a slightly narrower main lobe compared to the MV beamformer.

Additionally, the EMV presents the lower sidelobe levels compared to MV beamformer meaning that the EMV outperforms the MV beamformer in terms of lateral resolution by providing a lower FWHM and reduced PSL, respectively.

The GSC technique presented slightly narrower main lobe and lower side lobe level compared to EMV beamformer. The EGSC presented a narrower main lobe while lowering down the side lobe energy compared to GSC beamformer, which justifies the well-defined scatters on the displayed images.

Moreover, the CF-based methods came with remarkable improvements in image quality so that by combining the $\text {CF}_1$ with $\text {EGSC}$ resulted in the $\text {EGSC}_1$ beamformer which outperforms the $\text {EGSC}$ beamformer in terms of FWHM and PSL, respectively. Analogously, by combining the $\text {CF}_2$ with $\text {EGSC}$ we formulated the $\text {EGSC}_2$ beamformer which appears to outperform $\text {EGSC}_1$. Finally, on combining the $\text {CF}_3$ with $\text {EGSC}$ we formulated the $\text {EGSC}_3$ which in turn, outperforms $\text {EGSC}_2$, respectively.

Beyond the analysis of the displayed images, all the FWHM and PSL values are presented in Table 3. Remarkably, the $\text {EGSC}_3$ presented the improved lateral resolution by providing a narrower FWHM and lower PSL compared to the $\text {EGSC}_1$ and $\text {EGSC}_2$ so that the margins of difference in PSL (dB) were of 19.4 and 3, respectively. However, the FWHM was not as highly improved as the PSL meaning that the $\text {CF}_3$ appears to suppress more side lobes rather than narrowing the main lobe compared to the $\text {CF}_1$.

**Table 3:** Full width at half maximum and peak side lobe level results for simulated point targets for different beamformers

Beamformer	FWHM (mm)	PSL (dB)
DAS	1.73	− 23.4
MV	1.18	− 24.1
EMV	0.92	− 25.1
GSC	0.89	− 28.8
EGSC	0.78	− 29.9
$\text {EGSC}_1$	0.76	− 33.8
$\text {EGSC}_2$	0.69	− 51.4
$\text {EGSC}_3$	0.67	− 53.2

*FWHM* full width at half maximum,* PSL* peak side lobe level

Beyond the analysis of the displayed images, all the FWHM and PSL values are presented in Table 2. Remarkably, the $\text {EGSC}_3$ presented the improved lateral resolution by providing a narrower FWHM and lower PSL compared to the $\text {EGSC}_1$ and $\text {EGSC}_2$ so that the margins of difference in PSL were of 19.4 and 3 dB, respectively. However, the FWHM was not as highly improved as the PSL meaning that the $\text {CF}_3$ appears to suppress more side lobes rather than narrowing the main lobe compared to the $\text {CF}_1$.

### Simulation: circular anechoic cysts

**Fig. 8:**
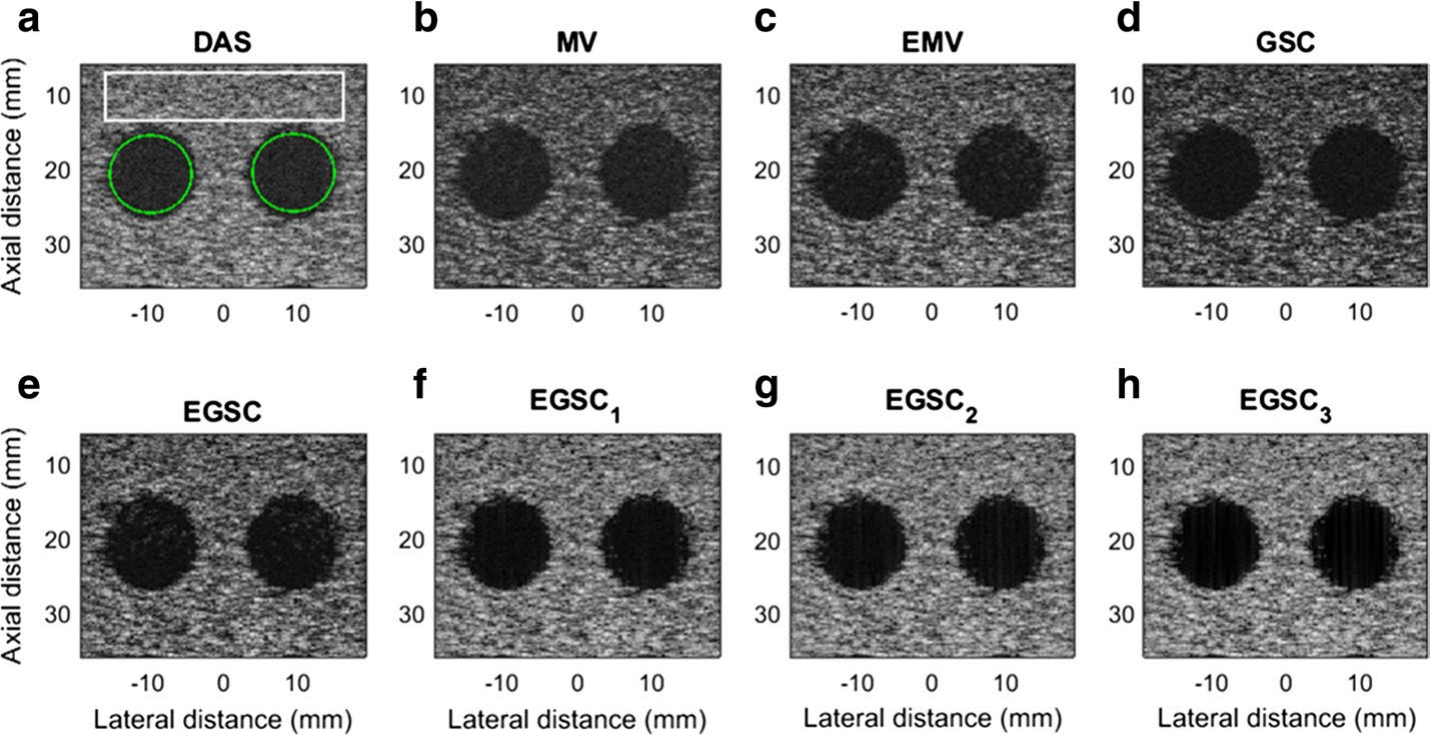
The beamformed responses for the simulated circular anechoic phantoms using different techniques: **a** DAS, **b** MV, **c** EMV, **d** GSC, **e** EGSC, **f** $\text {EGSC}_1$, **g** $\text {EGSC}_2$ and **h** $\text {EGSC}_3$, respectively

The simulated speckle pattern containing two circular anechoic cysts closely spaced was used for contrast and speckle statistics evaluation. For CR, CNR and SNR calculation, the background region marked in a white box and the interior of the cysts marked with green as shown in Fig. 8a were used for calculating the mean intensity in the background and inside the cyst, respectively. The responses for different techniques are displayed in Fig. 8.

In Fig. 8, it is shown that DAS, MV and GSC have a poor contrast due to higher side lobes level while the EMV and the EGSC can provide a better contrast. The EGSC with all versions of coherent factors has a relatively higher contrast and also exhibits clearly the cyst edge.

Quantitative results show that all the adaptive beamformers outperformed the DAS beamformer are presented in Table 4. Taking DAS as reference, the $\text {CR(dB)/CNR}$ presented the following improvements 1.5/0.08, 2.8/0.11, 3.0/0.16, 4.2/0.93, 6.1/1.07, 7.6/1.10 and, 9.1/1.12, for the MV, EMV GSC, EGSC, $\text {EGSC}_1$, $\text {EGSC}_2$, $\text {EGSC}_3$, respectively. In terms of percentage (%) improvement, the values are 5.9/5.36, 10.4/7.23, 11.1/10.19, 14.8/39.74, 20.2/43.14, 23.9/43.82, 27.4/44.26, respectively. As compared to $\text {EGSC}_1$ and $\text {EGSC}_2$, values of 1.5/0.03 and 3.0/0.05 were found representing in percentage (%) 4.7/1.19 and 9.0/1.97, respectively.

**Fig. 9:**
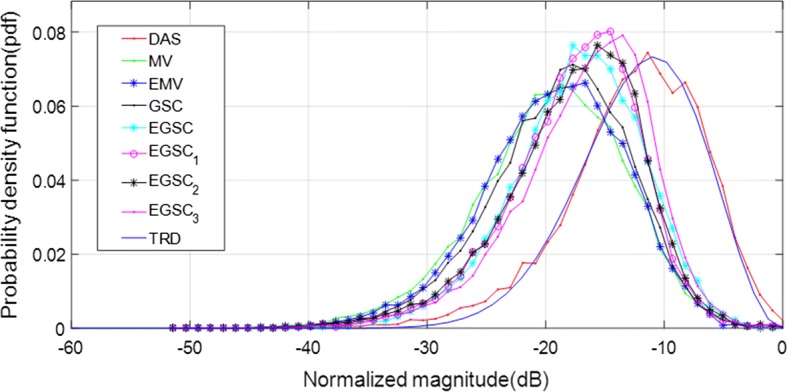
The speckle statistics of simulated data of Fig. 8 for different beamformers. The Theoretical Rayleigh distribution (TRD) fit to DAS is also included as the reference

Additionally, we have measured the speckle statistics for the different beamformers as presented in Fig. 9 complemented by different value of SNR presented in Table 4. For this purpose, the normalized pdf of the speckle region (see Fig. 8a white box) was computed for different beamformers.

Since the speckle pattern of DAS beamformer follows closely the TRD [7, 29], was included for illustration. All the beamformers were subject to the hypothesis test separately and the results showed that they followed the RD [7, 23, 29].

To complement the displayed images, the results are presented in Table 4. From Table 4, we can see that all the adaptive beamformers outperformed the DAS beamformer in terms of $\text {CR (dB)}/\text {CNR}$ with values of 1.5/0.08, 2.8/0.11, 3.0/0.16, 4.2/0.93, 6.1/1.07, 7.6/1.10 and, 9.1/1.12, were respectively obtained. These values represent in (%) values of 10.4/7.23, 5.9/5.36, 11.1/10.19, 14.8/39.74, 20.2/43.14, 23.9/43.82, 27.4/44.26, respectively. As compared to $\text {EGSC}_1$ and $\text {EGSC}_2$, values of 1.5/0.03 and 3.0/0.05 were found representing in percentage (%) 4.7/1.19 and 9.0/1.97, respectively.

**Table 4:** Contrast results for simulated circular anechoic cysts for different beamformers

Beamformer	IIC (dB)	IOC (dB)	CR (dB)	CNR	SNR
DAS	− 38.2	− 14.1	24.1	1.41	1.65
MV	− 40.1	− 14.5	25.6	1.49	1.74
EMV	− 42.4	− 15.5	26.9	1.52	1.64
GSC	− 46.6	− 19.5	27.1	1.57	1.56
EGSC	-48.1	− 19.8	28.3	2.34	1.48
$\text {EGSC}_{1}$	− 51.4	− 21.2	30.2	2.48	1.49
$\text {EGSC}_{2}$	− 52.2	− 20.5	31.7	2.51	1.56
$\text {EGSC}_{3}$	− 53.7	− 20.5	33.2	2.53	1.61

*IIC* intensity inside cyst,* CR* contrast,* IOC* intensity inside cyst,* SNR* signal to noise ratio

On observing Fig. 9, the MV, EMV, GSC and EGSC speckle pattern appears to be somewhat damaged compared to that presented by DAS so that the plots of the speckle patterns are slightly disparate. In GSC method, a slightly dark image can be seen in Fig. 8d which justifies the reduced SNR value, however, the $\text {EGSC}_1$, $\text {EGSC}_2$ and $\text {EGSC}_3$, respectively presented an increased SNR and hence, their corresponding plots of speckle pattern in Fig. 9 exhibited that their intensity levels in dB increased. This effect was in agreement with the SNR values presented in Table 4 in the sense that, among other beamformers, they were more similar compared to DAS beamformer. Table 7 presents the speckle magnitude of simulated data whose plots are shown in Fig. 9. The large values of speckle magnitude degradation for Fig. 9 in dB were − 20.0, − 16.2, − 17.3, − 17.3 for MV, EMV, GSC and EGSC respectively, contrary to − 14.8, − 15.7, − 13.8 for $\text {EGSC}_1$, $\text {EGSC}_2$ and $\text {EGSC}_3$, respectively. We notice that the speckle pattern of the EGSC-CF based methods is colse to that provided by DAS beamformer while providing improved lateral resolution and contrast.

### Experiments: phantom circular anechoic cyst

**Fig. 10:**
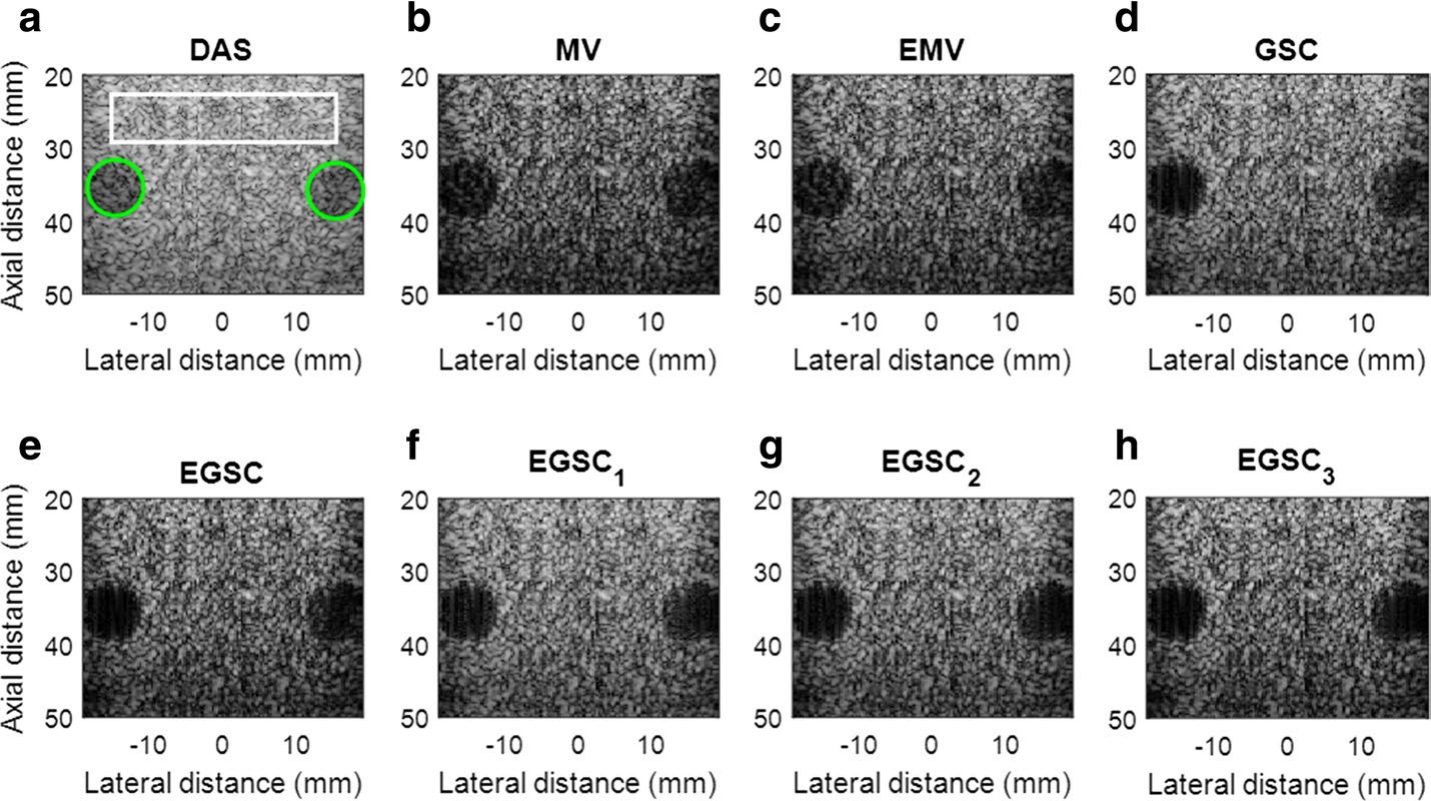
The beamformed responses of circular anechoic cysts using phantom data for different techniques: **a** DAS, **b** MV, **c** EMV, **d** GSC, **e** EGSC, **f** $\text {EGSC}_1$, **g** $\text {EGSC}_2$ and **h** $\text {EGSC}_3$, respectively

The cysts illustrated in Fig. 4 are ordered such that the rightmost cyst has − 9 dB contrast whereas, the cyst on the left has − 12 dB contrast, respectively. The images for the anechoic cyst using the various techniques are shown in Fig. 10.

For CR, CNR and SNR calculation, the background has been marked in a white box while the interior of the cysts was marked with the green circles as shown in Fig. 10a. These positions were used as the references for calculating the mean intensity in the background and inside the cyst, respectively.

The DAS, MV, and GSC exhibited a poor contrast due to higher side lobes level while the EMV and the EGSC can provide a better contrast than DAS, MV, and GSC, respectively, but the visibility of the edge of the cyst is still difficult. However, the EGSC with all versions of CF outperforms in terms of contrast and exhibits clearly the improvements of the cyst definition. Taking DAS as reference, the $\text {CR(dB)/CNR}$ presented the following improvements 1.5/0.18, 2.8/0.22, 4.0/0.31, 5.2/0.62, 7.1/0.84, 9.6/0.87 and, 10.1/0.90, for the MV, EMV GSC, EGSC, $\text {EGSC}_1$, $\text {EGSC}_2$, $\text {EGSC}_3$, respectively. In terms of percentage (%) improvement, the values are 5.9/10.1, 10.4/12.0, 14.2/16.1, 17.7/27.8, 22.8/34.3, 28.5/35.1 and, 29.5/35.9, respectively. As compared to $\text {EGSC}_1$ and $\text {EGSC}_2$, values of 2.5/0.03 and 3.0/0.06 were found representing in percentage (%) 7.4/1.2 and 8.8/2.4, respectively.

**Fig. 11:**
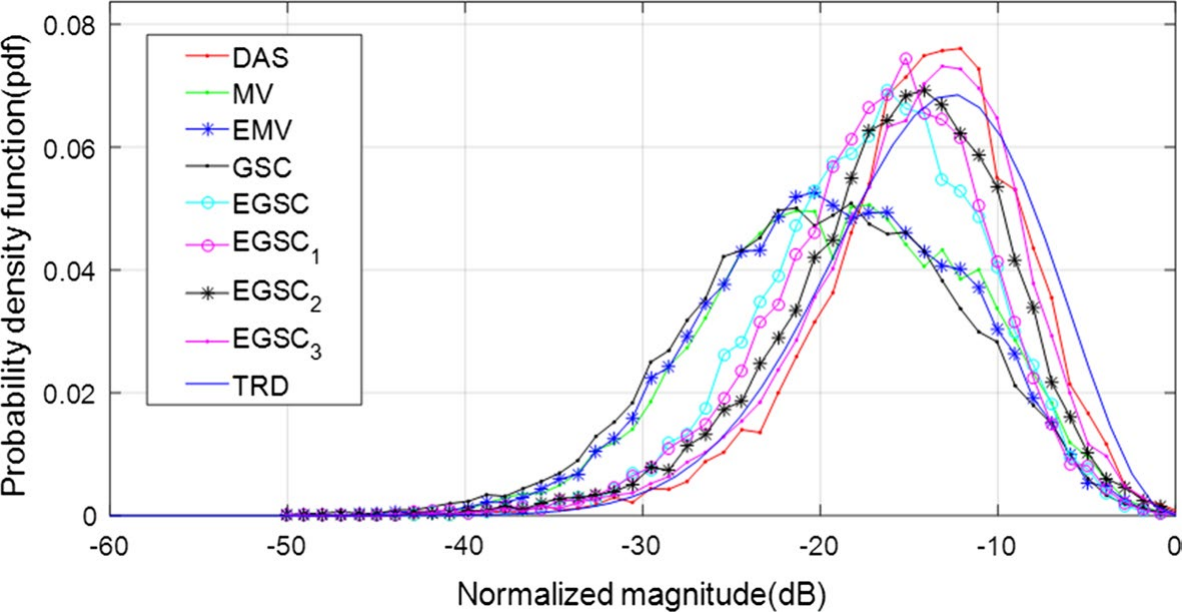
The speckle statistics of real data of Fig. 10 for different beamformers. The TRD fit to DAS is included as the reference

Additionally, we have measured the speckle statistics for the different beamformers as presented in Fig. 11 complemented by different value of SNR presented in Table 5. For this purpose, the normalized pdf of the speckle region (see Fig. 11a white box) was computed for different beamformers. Similarly to the simulated data, all the beamformers passed the hypothesis tests.

On observing Fig. 11, the MV, EMV, GSC and EGSC speckle pattern appears to be somewhat damaged compared to that presented by DAS so that the plots of the speckle patterns are slightly different. In GSC method, a slightly dark image can be seen in Fig. 10d which justifies the reduced SNR value, however, the $\text {EGSC}_1$, $\text {EGSC}_2$ and $\text {EGSC}_3$, respectively presented an increased SNR and hence, their corresponding plots of speckle pattern in Fig. 11 exhibited that their intensity levels in dB increased. This effect was in agreement with the SNR values presented in Table 5 in the sense that, among other beamformers, they were similar to DAS beamformer. Table 7 presents values of plots depicted in Fig. 11. The large values of speckle magnitude degradation for Fig. 11 in dB were − 21.3, − 20.0, − 19.0, − 16.6 for MV, EMV, GSC and EGSC respectively, contrary to − 15.2, − 14.4, − 13.6 for $\text {EGSC}_1$, $\text {EGSC}_2$ and $\text {EGSC}_3$, respectively. We remark that the speckle pattern of the EGSC-CF based methods is close to that provided by DAS while providing improved lateral resolution and contrast similarly to the simulated data.

**Table 5:** Contrast results for phantom circular anechoic cysts for different beamformers

Beamformer	IIC (dB)	IOC (dB)	CR (dB)	CNR	SNR
DAS	− 45.2	− 21.1	24.1	1.61	1.63
MV	− 47.1	− 22.6	25.6	1.79	1.65
EMV	− 48.4	− 22.5	26.9	1.83	1.61
GSC	− 49.6	− 22.5	28.1	1.92	1.58
EGSC	− 50.1	− 21.8	29.3	2.23	1.56
$\text {EGSC}_{1}$	− 50.4	− 21.2	31.2	2.45	1.58
$\text {EGSC}_{2}$	− 53.2	− 23.5	33.7	2.48	1.61
$\text {EGSC}_{3}$	− 55.7	− 25.5	34.2	2.51	1.74

*IIC* intensity inside cyst,* CR* contrast,* IOC* intensity inside cyst,* SNR* signal to noise ratio

### Experiments: phantom circular hyperechoic targets

**Fig. 12:**
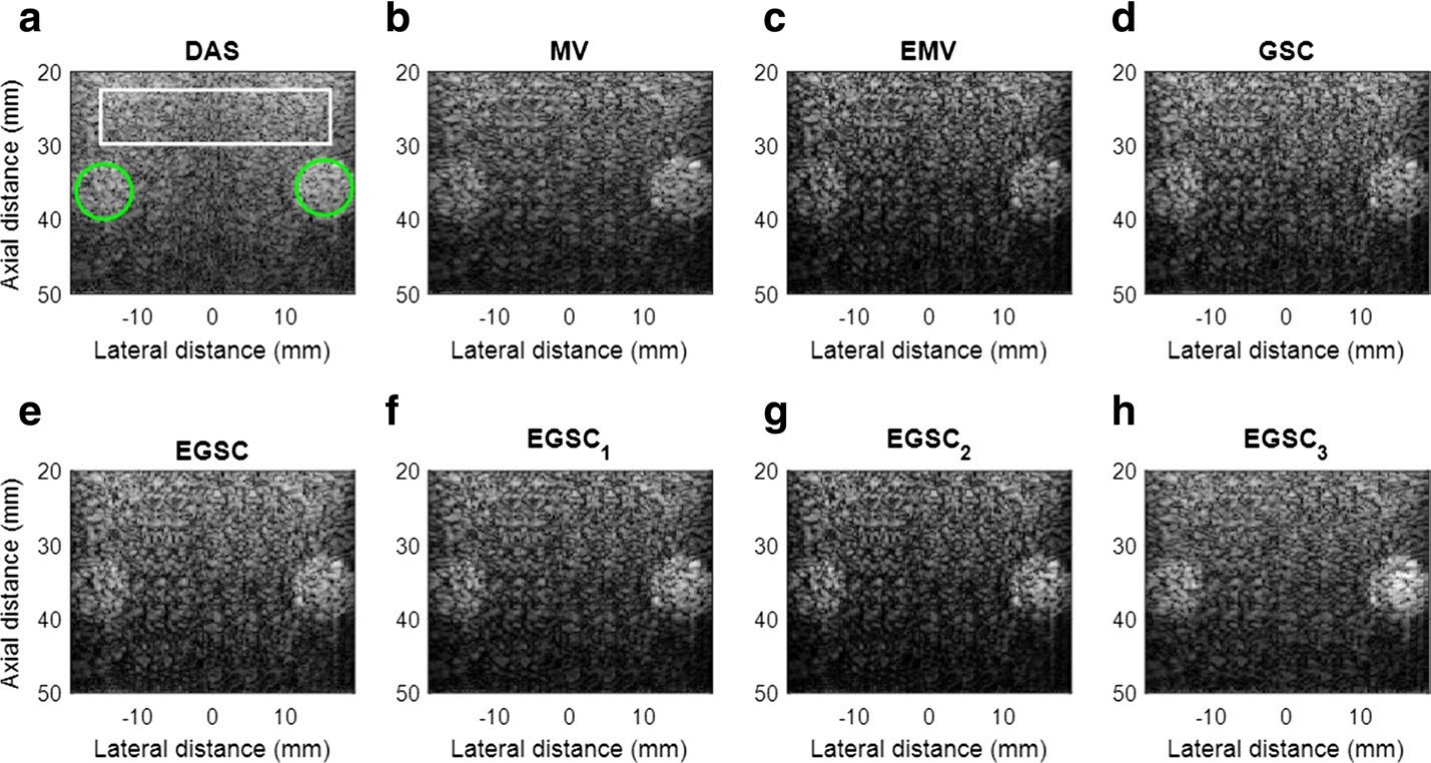
The beamformed responses of circular hypoechoic targets using phantom data for different techniques: **a** DAS, **b** MV, **c** EMV, **d** GSC, **e** EGSC, **f** $\text {EGSC}_1$, **g** $\text {EGSC}_2$ and **h** $\text {EGSC}_3$, respectively

Circular hyperechoic targets were also assessed and the images are shown in Fig. 12. For CR, CNR and SNR calculation, the background and the interior of the cysts was marked as shown in Fig. 12a.

The beamformed images reveal that the DAS, MV and GSC shows a poor contrast due to the higher side lobes level while the EMV and the EGSC provide an improved contrast compared to MV and the GSC.

The imaging contrast performance is quantified by using the contrast ratio (CR) and the analysis of the beamformed image data is done in terms of the quantitative values of intensities inside the target, intensities outside target, the CR, and the SNR as presented in Table 6.

**Table 6:** Contrast results for experimental circular hyperchoic cysts for different beamformers

Beamformer	IIC (dB)	IOC (dB)	CR (dB)	CNR	SNR
DAS	− 43.7	− 20.5	23.2	1.53	1.72
MV	− 45.2	− 20.7	25.5	1.68	1.74
EMV	− 48.4	− 22.2	27.2	1.74	1.68
GSC	− 49.4	− 22.1	27.3	1.85	1.62
EGSC	− 50.9	− 22.4	29.5	2.12	1.67
$\text {EGSC}_1$	− 52.1	− 22.7	30.4	2.26	1.67
$\text {EGSC}_2$	− 53.8	− 23.6	32.2	2.37	1.69
$\text {EGSC}_3$	− 55.2	− 23.9	33.3	2.53	1.71

*IIC* intensity inside cyst,* CR* contrast,* IOC* intensity inside cyst,* SNR* signal to noise ratio

Taking DAS as reference, the $\text {CR(dB)/CNR}$ presented the following improvements 2.1/0.15, 4.0/0.21, 4.1/0.32, 6.3/0.59, 7.2/0.73, 9.0/0.84 and, 10.1/1.0, for the MV, EMV GSC, EGSC, $\text {EGSC}_1$, $\text {EGSC}_2$, $\text {EGSC}_3$, respectively. In terms of percentage (%) improvement, the values are 8.3/8.9, 14.7/12.1, 15.0/17.3, 21.4/27.8, 23.7/32.3, 27.9/35.4 and, 30.3/39.5, respectively. As compared to $\text {EGSC}_1$ and $\text {EGSC}_2$, values of 2.9/0.27 and 1.1/0.16 were found representing in percentage (%) 8.7/10.7 and 3.3/6.3, respectively.

**Fig. 13:**
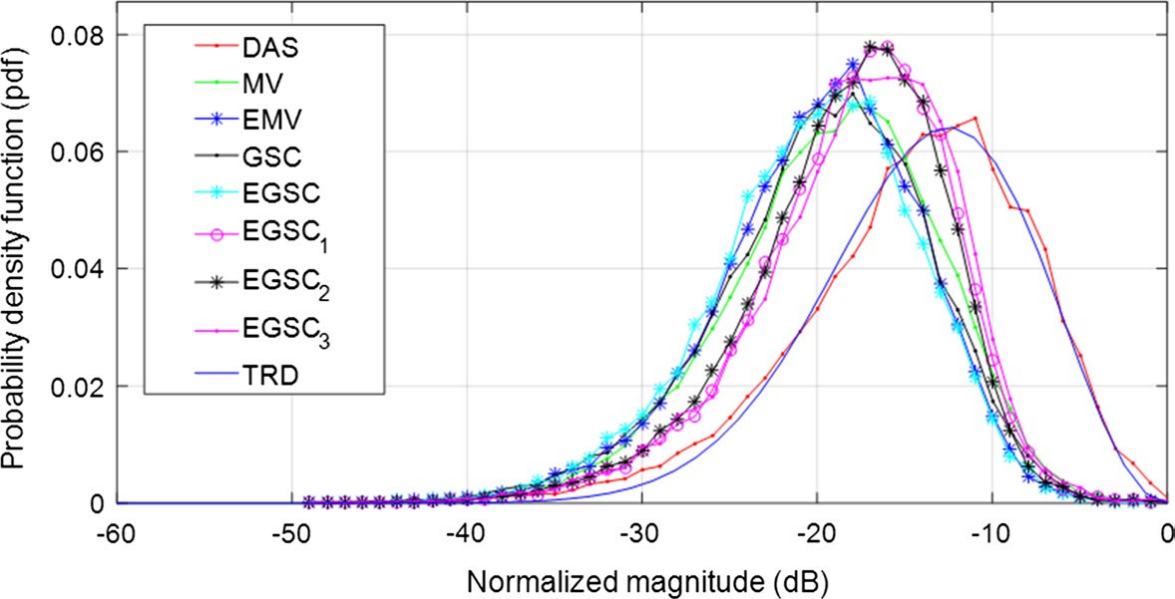
The speckle statistics of real data of Fig. 11 for different beamformers. The TRD fit to DAS is included as the reference

Additionally, the speckle statistics was evaluated as presented in Fig. 13 and Table 6. Similarly to the simulated data, all the beamformers passed the hypothesis tests. On observing Fig. 13, the MV, EMV, GSC and EGSC speckle pattern appears to be somewhat damaged compared to that presented by DAS so that the plots of the speckle patterns are slightly distinct.

In GSC method, a slightly dark image can be seen in Fig. 11d which justifies the reduced SNR value, however, the $\text {EGSC}_1$, $\text {EGSC}_2$ and $\text {EGSC}_3$, respectively presented an increased SNR and hence, their corresponding plots of speckle pattern in Fig. 13 exhibited that their intensity levels in dB increased. This effect was in agreement with the SNR values presented in Table 5 in the sense that, among other beamformers, they were relatively close compared to DAS beamformer.

Table 7 present values of plots depicted in Fig. 13. The large values of speckle magnitude degradation for Fig. 13 in dB were − 18.3, − 18.1, − 19.8, − 17.0 for MV, EMV, GSC and EGSC respectively, contrary to − 16.4, − 15.8, − 14.8 for $\text {EGSC}_1$, $\text {EGSC}_2$ and $\text {EGSC}_3$, respectively. We can see that the speckle pattern of the EGSC-CF based methods is close to that provided by DAS beamformer while providing improved lateral resolution and contrast.

**Table 7:** The average of magnitude (AM) of speckle for simulated and real data

Beamformer	Simulated AC anechoic cysts AM (dB)	Real data AC anechoic cysts AM (dB)	Real data HC hypoechoic targets AM (dB)
DAS	− 10.1	− 11.1	− 10.9
MV	− 20.0	− 21.3	− 18.3
EMV	− 16.2	− 20.0	− 18.1
GSC	− 17.3	− 19.0	− 19.8
EGSC	− 17.2	− 16.6	− 17.0
$\text {EGSC}_1$	− 14.8	− 15.2	− 16.4
$\text {EGSC}_2$	− 15.7	− 14.4	− 15.8
$\text {EGSC}_3$	− 13.8	− 13.6	− 14.8

### Computational complexity analysis

The main purpose of CPWC is to reduce the computational complexity (CC) aiming to increase the frame rate of the imaging system hence, the analysis of the CC is an important task. The indicative CC required to perform the DAS BF goes under *O*(*M*) where M is the array length set to be 128 and hence, it will need *O*(*M*) floating operations.

In adaptive methods the CC increases because the inversion and eigen decomposition operations of the CM. The CM inversion for MV require $2/3 \times \text {L}^3$ floating operations when applying Gauss-Jordan eliminations [4, 31] while the Hessian matrix inversion in Eq. (11) for GSC [17] needs $\text {L}^3$ floating operations. Note that the HM is more demanding than the CM. However, EMV and EGSC undergo the eigen decomposition of CM which in accordance with the Golub–Reinsch algorithm [32], need $21 \times \text {L}^3$ floating operations. Hence, the EMV will need $2/3 \times \text {L}^3+21 \times \text {L}^3$ while the EGSC $\text {L}^3+21 \times \text {L}^3=22 \times \text {L}^3$ [4, 17, 31].

For GCF computation in (16) an additional computational burden lead to the computation of the coherent and the incoherent energy [23] which is $\text {L}^2$. In the formulation of SCF ($\text {CF}_2$) Eq. (17) and the SNR-CF ($\text {CF}_3$) Eq. (19), the difference of the IS and CS is weighted using the scaling factors of $1/\text {L}$ and Eq. (18), respectively. In SNR-CF ($\text {CF}_3$) formulation, the weight $\eta (\text {SNR})$ Eq. (18) is more complex compared to $1/\text {L}$ in SCF ($\text {CF}_2$) formulation so that the computation of $\text {CF}_3$ will be more complex than $\text {CF}_2$.

In order to examine the processing flow, we have measured the time cost (in seconds) for each technique without any special implementation optimization in MatLab. The results are presented in Table 8 in terms of (mean ± standard deviation) reflecting the averaged time over 10 tests for simulated and the real data respectively, including the sizes of the reconstructed images. Computational time was measured for total image formation (i.e. for 21 steering angles for one image). For example, in accordance with Table 8, the proposed method increased the time cost compared to $\text {DAS}$, $\text {MV}$, $\text {EMV}$, $\text {GSC}$, $\text {EGSC}$, $\text {EGSC}_1$ and $\text {EGSC}_2$, respectively. For $\text {EGSC}_3$ compared to $\text {EGSC}_1$ and $\text {EGSC}_2$ the time in percentage (%) increased with the following values 7.0, 5.0 and 11.6, 6.5 for point targets and cyst simulation and, 13.5, 11.0 and 11.8, 8.2 for real data involving the anechoic cysts and the hyperchoic targets, respectively.

**Table 8:** Time cost (in seconds) for all beamformers for simulated and real data

Beamformer	Simulated PT	Simulated AC	Real data AC	Real data HC
	Size (1620 $\times$ 128)	Size (1526 $\times$ 128)	Size (2048 $\times$ 128)	Size (2048 $\times$ 128)
DAS	109.1 ± 0.21	120.2 ± 0.23	133.2 ± 0.13	123.6 ± 1.23
MV	621.3 ± 0.31	792 ± 0.31	675.0 ± 0.22	732.6 ± 0.32
EMV	1075.2 ± 0.26	7236.1 ± 0.41	1260.6 ± 0.91	1239.0 ± 0.60
GSC	798.7 ± 0.31	864.8 ± 0.53	739.2 ± 0.43	795.6 ± 0.51
EGSC	1234.8 ± 0.22	1332.6 ± 0.23	1328.4 ± 0.51	1456.2 ± 0.4
$\text {EGSC}_1$	1432.2 ± 0.33	1584.0 ± 0.14	1464.6 ± 0.64	1506.0 ± 0.22
$\text {EGSC}_2$	1465.8 ± 0.25	1638.4 ± 0.32	1506.0 ± 0.32	1567.8 ± 0.08
$\text {EGSC}_3$	1543.2 ± 0.45	1752.2 ± 0.33	1674.6 ± 0.31	1708.2 ± 0.43

*PT* point target,* AC* anechoic cyst,* HT* hypoechoic target

Among the CF based methods, the processing flow indicated values of time somewhat close one another however, there was an agreement with the indicative CC analysis.

In general, the CF-based methods increase the computational complexity so that the $\text {EGSC}_3$ method will be more time demanding than $\text {EGSC}_2$, and $\text {EGSC}_2$ more time consuming than the $\text {EGSC}_1$ technique. Therefore, the improvements in image quality introduced by the EGSC-CF beamformers were at the cost of an extra computational load compared to the different adaptive methods. For real-time applications, the most significant computational complexity added (i.e., the matrix inversion computation of the CM or HM) can be executed applying appropriate algorithms and architectures such as recursive updating [14], and graphics processing units [4], respectively.

## Conclusion

The eigenspace generalized sidelobe canceller EGSC beamformer combined with the SNR dependent coherence factor SNRCF method has been proposed for coherent plane wave compounding imaging. The technique, here called $\text {EGSC}_3$ beamformer was compared with various adaptive beamformers (i.e., MV, EMV, GSC and EGSC) and other coherence factor based adaptive beamformers such as generalized coherence factor (GCF) and subarray coherence factor (SCF) using simulated and experimental data.

Taking DAS as reference, $\text {EGSC}_3$ showed improvements of 30.3 and 39.5% for $\text {CR (dB)}$ and $\text {CNR}$, respectively, using experimental data. As compared to $\text {EGSC}_1$ and $\text {EGSC}_2$, improvements of 2.9 and 1.1% for $\text {CR (dB)}$ and 0.27 and 0.16 in $\text {CNR}$ were found, respectively. Even thought the image quality improvement compared to other EGSC methods is small, the values of speckle statistics of $\text {EGSC}_3$ outperforms the $\text {EGSC}_1$ and $\text {EGSC}_2$ methods and remained close to DAS contrary to the remaining adaptive beamformers. The $\text {EGSC}_3$ is, therefore, suitable for CPWC by improving the spatial resolution and contrast while preserving the speckle pattern different from those beamforming methods that do not use coherence factors.
